# Sigmoid colon translocation of an intrauterine device misdiagnosed as a colonic polyp: A case report

**DOI:** 10.1097/MD.0000000000009840

**Published:** 2018-02-09

**Authors:** Xin-Xin Zhou, Mo-Sang Yu, Meng-Li Gu, Wei-Xiang Zhong, Hong-Ru Wu, Feng Ji, Hang-Hai Pan

**Affiliations:** a Department of Gastroenterology; b Department of Pathology, The First Affiliated Hospital, College of Medicine, Zhejiang University; c Department of Gastroenterology, Zhejiang Greentown Cardiovascular Hospital; d Department of Gastroenterology, Zhejiang Provincial People's Hospital, Hangzhou, Zhejiang Province, China.

**Keywords:** chronic perforation, colonic polyp, intrauterine contraceptive devices, translocation of intrauterine contraceptive device

## Abstract

**Rationale:**

Intrauterine contraceptive devices (IUDs) are recommended as a means of contraception. Translocation of IUD is a rare and serious complication. Colonic inflammatory mass caused by translocated IUD initially misdiagnosed as a colonic polyp is extremely rare and has not been reported yet.

**Patient concerns:**

This report presents a case of sigmoid colon translocation of intrauterine device on a 37-year-old female patient. Colonoscopy was performed due to her complain of repeated blood in stools and subsequently the patient was misdiagnosed as a sigmoid colon polyp. Nonetheless, the “polyp” was not able to be removed endoscopically.

**Diagnoses:**

Sigmoid colon translocation of an intrauterine device.

**Interventions:**

To further clarify the diagnosis, computed tomography (CT) scan was performed and the “polyp” was confirmed to be caused by a translocated IUD.

**Outcomes:**

The translocated IUD was removed easily by surgery, and the patient recovered soon after the operation.

**Lessons:**

The present case indicates that an annual gynaecologic examination is necessary to determine the position of the IUD, and a CT examination may help confirm an ectopic IUD.

## 1 Introduction

Intrauterine contraceptive devices (IUDs) are a safe, effective, reversible, and permanent method for contraception and are recommended as a first-line contraception by the World Health Organization (WHO) and the Centers for Disease Control and Prevention (CDC) in America.^[1,2]^ Fewer than 1 in 100 women become pregnant during the first year of IUD use.^[2,3]^ IUDs are also commonly used to prevent unintended pregnancies in China. The major complications of IUDs are displacement and unsuccessful retention.^[4]^ A displaced IUD may lead to perforation of the uterus and further damage to adjacent viscera such as the intestines and urinary bladder.^[5–7]^

Herein, we first report a rare case of sigmoid colon translocation of IUD following its penetration of the uterine wall, leading to local inflammatory responses and further formation of inflammatory granulation tissue, which is misdiagnosed as a colonic polyp.

## 2 Case report

**Figure 1:**
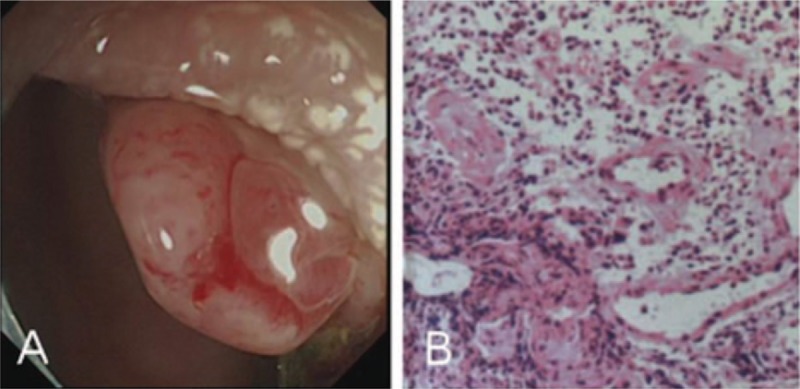
(A) A “polyp” was found in sigmoid colon under endoscopy. (B) Pathological findings indicated that the “polyp” consisted mainly of inflammatory granulation tissue (HE staining,  × 100).

A 37-year-old female patient presented with a half-year history of recurrent hematochezia. Half a year ago, the patient complained about bloody stool, particularly after eating spicy food. She then made an appointment with her local hospital for a detailed examination. When questioned directly, the patient denied any abdominal pain, changes in vaginal secretions, vaginal bleeding, or pain during sex or urination. A colonoscopy examination revealed a “polyp” with a diameter of 10 mm in the sigmoid colon (Fig. 1A). There was a dark scar, which may be caused by the bleeding, at the base of the “polyp.” A biopsy was performed, and the loose tissue was prone to bleeding. A titanium clip was used to prevent hemorrhaging. Pathologic results indicated that the “polyp” consisted mainly of inflammatory granulation tissue (Fig. 1B). A month later, the patient returned to the local hospital to remove the “polyp.” However, the excision of the “polyp” was unsuccessful, and the titanium clip was left in the colon.

**Figure 2:**
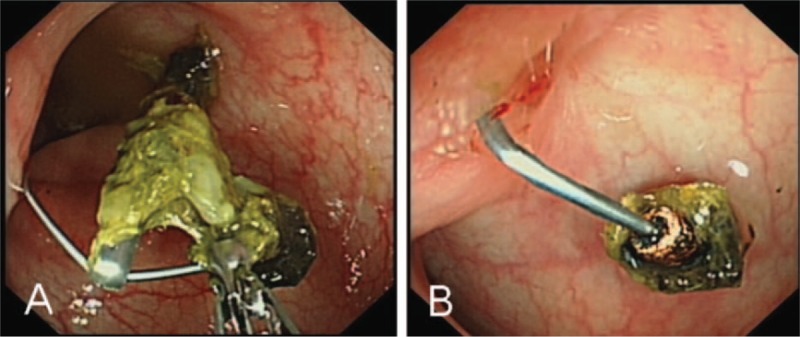
(A) The “polyp” disappeared and the titanium clip remained in the colon. (B) A metal object was observed when the clip was removed.

**Figure 3:**
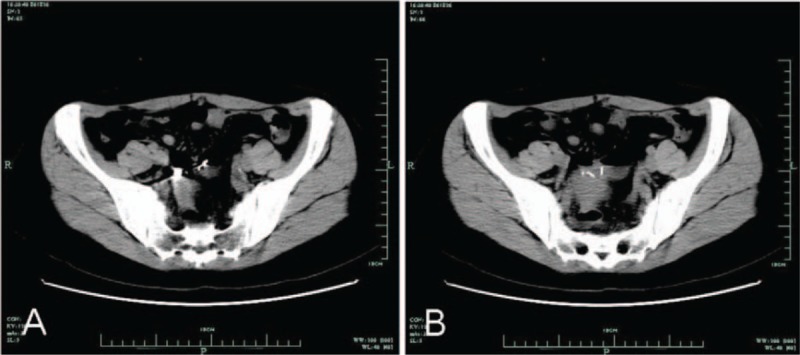
(A, B) Computed tomography reveals an IUD translocated to the sigmoid colon.

After half a year, the patient came to our hospital for a review and colonoscopy was performed. Surprisingly, the “polyp” disappeared and the titanium clip remained in the colon (Fig. 2A). Biopsy forceps were used to pick off the titanium clip and a metal object was unexpectedly observed when the clip was removed (Fig. 2B). This metal object was further verified as a translocated IUD by abdominal computed tomography (CT) scan (Fig. 3 A,B). Therefore, we carefully asked about her medical history and found that she had a uterine IUD implanted for contraception at 6 months postpartum 2 years ago. However, she got pregnant again without removing the IUD and the ultrasound examination showed no evidence of the IUD. Then, the disappeared IUD was not found during her subsequent induced abortion as well. Nevertheless, the patient paid little mind to this, as she did not experience obvious discomfort and she never anticipated that the IUD was causing her recurrent hematochezia.

**Figure 4:**
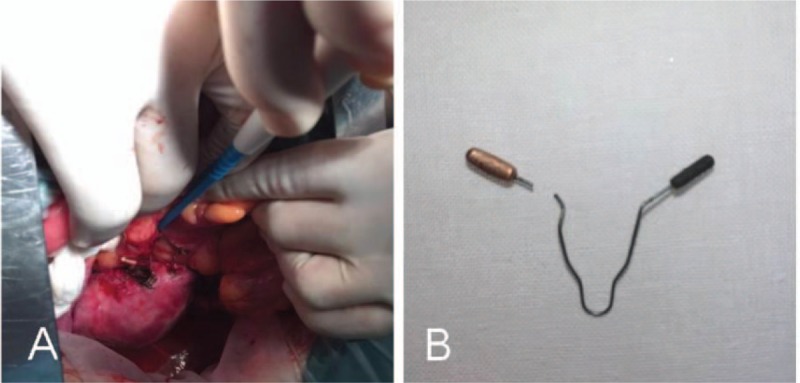
(A) An IUD was found penetrating the uterine wall and removal of the IUD by surgery. (B) Removed intrauterine device.

After thorough examinations, the patient was transferred to a surgeon for another operation. During this operation, the skin was incised along the ventral midline. The surgeon opened the abdomen and examined it carefully, locating an adhesion of the uterus to the sigmoid. After detaching the adhesion, the chief surgeon observed that the 2 arms of the IUD had penetrated the uterine wall (Fig. 4A). One end was perforated into the colon, and the other end was hidden in the mesentery. After one expanded end of the IUD was cut off, the IUD was removed (Fig. 4B) and the perforations of the colon wall and uterus were repaired carefully. The patient recovered well after the operation and returned home on postoperative day 5 with no complications.

## 3 Discussion

IUD is a safe, effective, convenient method for contraception. Given that the WHO recommends waiting 24 months after birth before attempting a subsequent pregnancy, the use of intrauterine contraception soon after childbirth has become increasingly popular.^[8]^ First, it eliminates the need for a return visit to start contraception. Moreover, a longer birth interval benefits the mothers’ and infants’ health and reduces maternal complications greatly. In addition, an IUD is long-acting and reversible, which is more appropriate for those who seek long-term contraception.^[2]^ However, the postnatal uterus is always soft and thin-walled, leading to potential IUD expulsions and displacements.

Uterine perforation after insertion is a rare complication of IUDs with an incidence of 1.3 to 1.6/1000.^[9]^ Generally, the perforation occurs through the posterior wall of the uterus. Following perforation, the misplaced IUDs are often found in several adjacent organs. The translocation of IUD in gastrointestinal tract occurred mostly in sigmoid colon (40.4%), and then in small intestine (21.3%) and rectum (21.3%).^[10–12]^ Previous literatures have reported migration of IUD may cause serious complications such as gastrointestinal perforation, intestinal obstruction, fistula, intra-abdominal abscess, and peritonitis.^[9,11,13]^ However, colonic inflammatory mass caused by translocated IUD initially misdiagnosed as a colonic polyp is extremely rare and has not been reported yet. The present case shows a polypoid change in colon caused by inflammatory responses following a translocated IUD penetrated the colon wall. As there was no sign of acute perforation and the inflammatory change caused by translocated IUD is similar to colonic polyps, it is easy to be misdiagnosed.

The pathological types of colonic polyps include adenomatous polyps, inflammatory polyps, haematoma polyps, hyperplastic polyps, and carcinoid polyps.^[14]^ Inconsistent with the clinical features and endoscopic manifestation, the pathological results of this case indicated inflammatory granulation tissue. Thus, it requires more caution when such discrepancies exist. Furthermore, it is of great importance to carefully ask medical history in detail.

Numerous factors will lead to IUD displacement and incarceration. The following 2 congenital factors may contribute to the incidence of ectopic IUDs: uterine malformation and uterine enlargement. Other possible contributions include postpartum, postabortion, lactation, and metratrophia. Mismatched size, strenuous exercise, and psychological factors may also influence the IUD's position. Eichengreen et al^[15]^ once reported that a 27-year-old woman, who had an IUD placed at a 6-week postpartum visit, suffered a rectal perforation 1 year after the IUD was implanted. In our case, the patient's IUD was inserted at 6 months postpartum during the lactation period, which may be a risk factor for IUD displacement and uterine perforation.^[11]^ In addition, the posterior uterus also increased the risk of IUD displacement.^[16]^

The primary symptoms of ectopic IUD may include pain at the time of insertion, delayed abdominal or pelvic pain, and irregular vaginal bleeding.^[17]^ However, many women with extrauterine IUDs are asymptomatic, and few cases are recognized at the time of IUD insertion. Most patients who come to the hospital complain of a missing IUD.^[18]^ Considering the chronical development of IUD penetration to the colon wall, our case merely displays minor recurrent blood in stools without any other symptoms.

Ultrasound examination is a fast and noninvasive way to evaluate uterine conditions and diagnose IUD displacement. Ultrasound examination after IUD insertion may be an effective way to predict the success of IUD retention. The IUD is defined as being “in place” when visualized in close proximity to the uterine fundus and the distance from each uterine wall to the body of the IUD is similar.^[4]^ Finally, if ultrasound examination cannot locate the IUD, a CT scan will help.

For women who have IUDs implanted, an annual gynecological examination is recommended to check the position of the IUD.^[2]^ Once acute abdominal pain, irregular vaginal bleeding, or bloody stool occurs, an ultrasound examination can assist in distinguishing ectopic IUDs from other causes. If no IUD is found in the uterus, a supplementary abdominal CT examination can assist with correct diagnosis, and a thorough examination is certainly helpful. The patient in the present case neglected the absence of the IUD and did not undergo further examination, leading to the delay of “timely” decisions regarding her treatment.
